# One-pot synthesis of MWW zeolite nanosheets using a rationally designed organic structure-directing agent†
†Electronic supplementary information (ESI) available: Experimental procedures, tables of synthesis screen results, ^1^H and ^13^C liquid and MAS NMR spectra of OSDA, ^31^P MAS NMR spectra of TMPO and TBPO titrations. See DOI: 10.1039/c5sc01912e


**DOI:** 10.1039/c5sc01912e

**Published:** 2015-07-22

**Authors:** Helen Y. Luo, Vladimir K. Michaelis, Sydney Hodges, Robert G. Griffin, Yuriy Román-Leshkov

**Affiliations:** a Department of Chemical Engineering , Massachusetts Institute of Technology , Cambridge , MA 02139 , USA . Email: yroman@mit.edu ; Tel: +1-617-253-7090; b Department of Chemistry and Francis Bitter Magnet Laboratory , Massachusetts Institute of Technology , Cambridge , MA 02139 , USA

## Abstract

A new material MIT-1 comprised of delaminated MWW zeolite nanosheets is made in a one-pot synthesis using a rationally designed organic structure-directing agent (OSDA). The OSDA consists of a hydrophilic head segment that resembles the OSDA used to synthesize the zeolite precursor MCM-22(P), a hydrophobic tail segment that resembles the swelling agent used to swell MCM-22(P), and a di-quaternary ammonium linker that connects both segments. MIT-1 features high crystallinity and surface areas exceeding 500 m^2^ g^–1^, and can be synthesized over a wide synthesis window that includes Si/Al ratios ranging from 13 to 67. Characterization data reveal high mesoporosity and acid strength with no detectable amorphous silica phases. Compared to MCM-22 and MCM-56, MIT-1 shows a three-fold increase in catalytic activity for the Friedel–Crafts alkylation of benzene with benzyl alcohol.

## Abstract 2

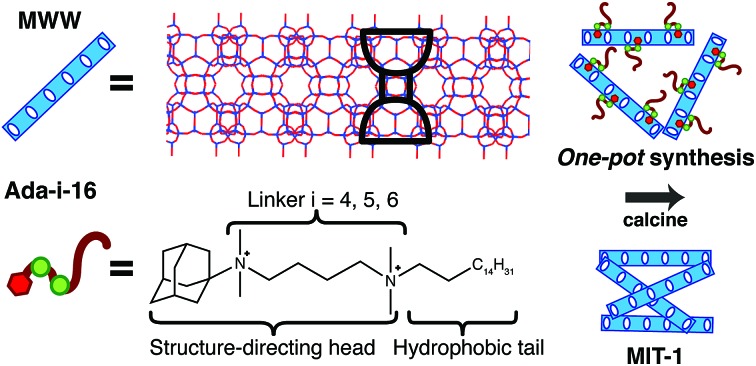
A new material MIT-1 comprised of delaminated MWW zeolite nanosheets is synthesized in one-pot using a rationally designed organic structure-directing agent.

## 

In recent years, layered zeolite precursors have garnered increased attention as a platform for developing new materials.^1^–^3^ Through post-synthetic modifications, these layered zeolite precursors can be transformed into 2-dimensional (2D), zeolites with open architectures. These novel hierarchical microporous/mesoporous materials with exposed active sites can facilitate the conversion of bulky substrates while maintaining higher stability than amorphous mesoporous materials.^4^–^7^ An important aluminosilicate layered zeolite precursor is MCM-22(P)^8^,^9^ (isostructural to SSZ-25,^10^,^11^ ERB-1,^12^,^13^ and PSH-3 14,15), which forms in single unit-cell thick (*ca.* 2.5 nm) layers with the MWW topology. These layers are arranged perpendicular to the *c*-axis such that half of the 12-ring cage is exposed to the crystal exterior, effectively forming “cups” of fully connected tetrahedral atoms on each side of the layer (see Scheme 1).^16^ In contrast to typical surface acid sites, the Brønsted acid sites located in the cups are as strong as those located inside micropores.^17^,^18^ Unfortunately, upon calcination, the layers of MCM-22(P) condense topotactically to form the microporous three-dimensional (3D) zeolite MCM-22 (12-ring cages connected by 10-ring channels).

**Scheme 1:**
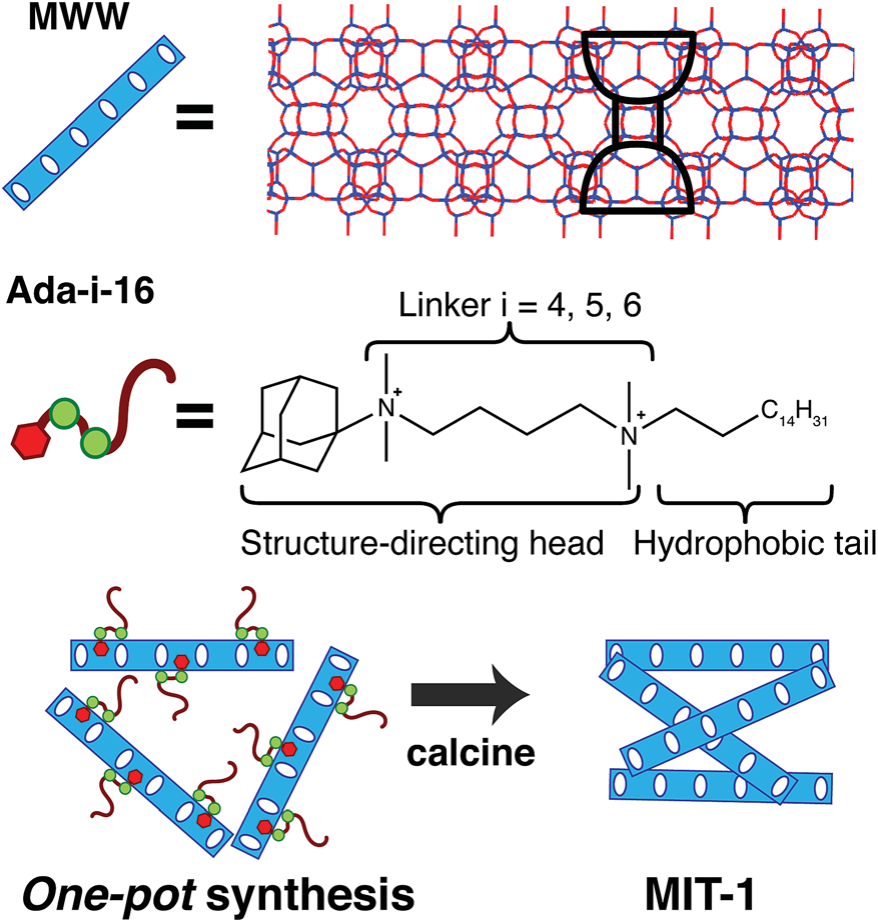
Schematic representation of the one-pot synthesis strategy to create MIT-1.

As such, post-synthetic methods have been developed to prevent layer condensation and generate exfoliated MWW nanosheets with a large fraction of exposed cups. Corma *et al.* developed ITQ-2 by swelling the layers of MCM-22(P) with a quaternary ammonium surfactant and then delaminating the swollen sheets by ultrasonication.^19^ The calcined material, comprised of disordered sheets, featured very high external surface areas of *ca.* 700 m^2^ g^–1^ and was shown to be active for the cracking of vacuum gas oil,^19^ decalin and tetralin,^20^ as well as the isomerization of *m*-xylene.^21^ Exfoliation was shown to be most effective over a specific set of conditions that include using highly alkaline conditions (pH > 12.5 at 353 K)^21^ and precursor materials with Si/Al ratios >20.^22^ In search of less damaging post-synthetic treatments, Maheshwari *et al.* demonstrated that the swelling step could be done at room temperature,^23^ and Maluangnont *et al.* produced a stable colloidal suspension of MWW monolayers without ultrasonication.^24^ Varoon *et al.* synthesized highly crystalline MWW nanosheets by melt blending layered precursors to produce polystyrene nanocomposites,^25^ while Ogino *et al.* exfoliated MCM-22(P) layers using surfactants at pH = 9 without ultrasonication, albeit producing sheets with lower mesoporosity than ITQ-2.^26^ For borosilicates, Ouyang *et al.* achieved a single-step delamination and isomorphous substitution of B with Al by treating ERB-1 with an aqueous aluminum nitrate solution at 408 K.^27^ However, the resulting material required a final dealumination step by acid treatment to remove extra-framework species.

Although one-pot synthesis methods are preferable for process intensification, they have been largely unsuccessful in creating materials with comparable properties to those obtained with multi-step, post-synthetic methods. For example, zeolites MCM-56,^28^–^30^ ITQ-30,^31^ and EMM-10 32 exhibit disorder in the stacking of layers perpendicular to the *c*-axis, but their low mesoporosity indicates their structure more closely resembles their 3D counterparts. Although very careful control of the MCM-56 synthesis has yielded a possible unilamellar structure, the material must be delaminated or pillared to maintain high external surface areas after calcination.^33^,^34^ Consequently, the development of an effective and robust method to create high-quality MWW nanosheets without additional post-synthetic treatments continues to be challenging.

Here, we demonstrate an effective one-pot synthesis method to generate exfoliated single-unit-cell thick MWW nanosheets. The new material, named MIT-1, is synthesized using a rationally-designed OSDA and results in a material with high crystallinity, surface area, and acidity that does not require post-synthetic treatments other than calcination. The OSDA is comprised of a diquaternary ammonium surfactant with tailored structure-directing head, alkyl linker, and hydrophobic tail groups that direct the formation of the MWW topology (see Scheme 1). A parametric study of Al, Na, and water content reveals that MIT-1 crystallizes over a wide synthetic window. Characterization data show that MIT-1 has high mesoporosity with an external surface area exceeding 500 m^2^ g^–1^ and a high external acid site density of 21 × 10^–5^ mol g^–1^. Catalytic tests demonstrate that MIT-1 has three-fold higher catalytic activity for the Friedel–Crafts alkylation of benzene with benzyl alcohol as compared to that of MCM-22 and MCM-56.

The strategy to design an OSDA that could produce MWW nanosheets in one-pot is depicted in Scheme 1. Inspired by the recent work by Ryoo *et al.*,^35^ we surmised that a suitable OSDA should combine the elements of the traditional OSDA used for the synthesis of the MWW layered zeolite precursor and the quaternary ammonium surfactant typically used for the swelling step during post-synthetic delamination. MCM-22(P) can be synthesized using hexamethyleneimine (HMI) or trimethyladamantylammonium hydroxide (Ada-OH), while swelling is typically performed with hexadecyltrimethylammonium bromide (CTAB). As shown in Scheme 1, the novel OSDA, named Ada-*i*-16 (where *i* = 4, 5, or 6 –CH_2_– linker groups), has a hydrophobic tail segment that resembles CTAB, a hydrophilic head segment that resembles Ada-OH, and a di-quaternary ammonium linker that connects both segments. The linker’s ammonium composition and chain length were tuned to achieve an effective C/N^+^ ratio ranging from 17–18, which is close to the optimal values (i.e., 10–15) previously identified for high silica hydroxide syntheses,^36^,^37^ and which decreases the risk of solubility problems for the OSDA in water. We note that previous attempts to use surfactants with single ammonium moieties to crystallize zeolites have only resulted in ordered amorphous materials.^38^,^39^ We varied the linker size between 4 and 6 –CH_2_– units because the linker size can affect the mobility and interdigitation of the C_16_ tails, thereby influencing the packing (*i.e.*, unilamellar *vs.* multilamellar) of the layers.^40^ Molecular dynamics simulations indicate that the structure-directing head sits inside of the cups with the diquaternary ammonium moieties stabilizing the pore mouth (ESI, Fig. S1†). Details of the procedure to synthesize Ada-*i*-16 can be found in the ESI.†

An initial screening to understand the effect of Ada-*i*-16 composition on MIT-1 crystallization was performed using a synthesis gel of 1 SiO_2_/0.1 Ada-*i*-16/0.05 Al(OH)_3_/0.2 NaOH/45 H_2_O at 433 K with rotation at 60 rpm. This gel composition resembles the one typically used to make MCM-22(P); while the Ada-*i*-16/Si ratio of 0.1 is similar to that used for the synthesis of MFI nanosheets with similar diquaternary surfactants.^40^ Varying the linker size drastically affected the crystallization time (Table S1, ESI†). Specifically, fully crystalline MIT-1 was obtained in 14 and 22 days when using Ada-4-16 and Ada-6-16, respectively. Interestingly, the C_5_ linker did not yield a crystalline product even after 30 days. ^13^C magic-angle spinning nuclear magnetic resonance (MAS NMR) on the as-synthesized material confirms that the OSDA remains intact in the pores (see Fig. S2–S6, ESI†). Increasing the Ada-4-16/Si ratio up to 0.3 did not affect the synthesis time or phase purity. Decreasing the temperature from 433 K to 423 K doubled the synthesis time, but did not alter the product phase. Adding Ada-OH as a co-template at Ada-OH/Ada-4-16 ratios ranging from 0.025 to 1 promoted the crystallization of 3D SSZ-13 (CHA topology) after short times. Indeed, Ada-OH is a well-known OSDA for the synthesis of SSZ-13 at these temperatures and gel compositions.^41^ At even lower Ada-OH contents, the co-template did not have a noticeable effect, and the MIT-1 phase was observed exclusively.

The powder X-ray diffraction (PXRD) patterns acquired after calcination of MIT-1 (synthesized with Ada-4-16) at 813 K for 10 h confirm that the sample has the MWW topology (Fig. 1A). The diffraction pattern features broader peaks than those observed for MCM-22. More specifically, the pattern shows reflections belonging to the (*hk*0) directions, indicating the absence of long-range order in the *c*-direction, as expected for exfoliated MWW layers.^42^ Simulated diffraction patterns (obtained using powder pattern theorem for ultrasmall zeolite crystals implemented with UDSKIP)^43^,^44^ for MWW crystalline constructs that are 15 unit cells wide along the *a*- and *b*-axes and one unit-cell thick along the *c*-axis are in good agreement with the experimental PXRD patterns for MIT-1. Extending the crystallization time by seven additional days yields a material with additional diffraction peaks, consistent with the mordenite (MOR) topology as well as increased condensation of the nanosheets into the 3D MWW structure as confirmed by transmission electron microscopy (TEM) (Fig. S7 and S8, ESI†). MOR impurities have been previously observed in MCM-22(P) syntheses,^45^ and condensation of disordered sheets into a multilamellar structure has been observed for MFI nanosheets crystallized for longer periods of time.^46^

**Fig. 1:**
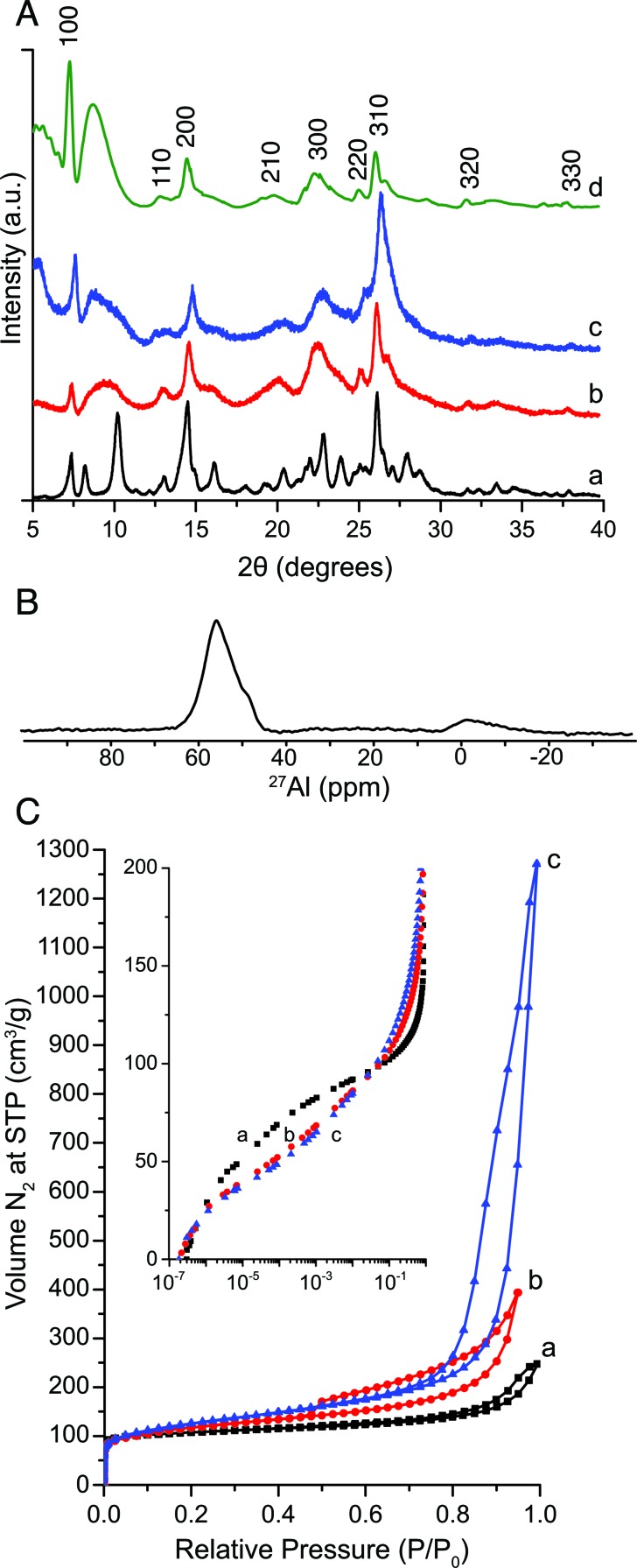
PXRD patterns (A) for calcined MCM-22 (a), MCM-56 (b), MIT-1 (c), simulated MWW nanosheets (d). ^27^Al MAS NMR spectra of as-synthesized MIT-1 (B). N_2_ adsorption and desorption isotherms (C) for calcined MCM-22 (a), MCM-56 (b), MIT-1 (c). Inset shows data on a semi-log plot.

Scanning electron microscopy of MIT-1 reveal particles composed of disordered platelets agglomerated into >10 μm clusters (Fig. 2). No other morphologies were detected during low magnification inspections. TEM confirmed the presence of disordered nanosheets *ca.* 2.5 nm thick along the (001) direction and *ca.* 150 nm (spanning 50–200 nm) long along the (100) and (010) directions. Selected area electron diffraction perpendicular to the plane of the sheets (inset, Fig. 2) reveals the expected hexagonal symmetry of MWW topology crystals. Nitrogen adsorption studies demonstrate that MIT-1 has much higher mesoporosity than MCM-22 or MCM-56 with a very broad mesopore size distribution (see Fig. 1C and S9 ESI†). The total pore volume and external surface area of MIT-1 after calcination are 1.014 cm^3^ g^–1^ and 513 m^2^ g^–1^, respectively (see Table S2 and Fig. S10, ESI†). This surface area is very close to the theoretical value of 517 m^2^ g^–1^ calculated for 150 × 150 nm long and 2.5 nm thick MWW sheets using geometric arguments (see Table S3, ESI†). In contrast, the total pore volume and external surface area of MCM-22 are three times lower at 0.289 cm^3^ g^–1^ and 121 m^2^ g^–1^, respectively. For MCM-56, the total pore volume and external surface area are two times lower at 0.601 cm^3^ g^–1^ and 219 m^2^ g^–1^, respectively. A log-plot of the adsorption isotherms (Fig. 1C, inset) shows that, in the pressure range of 10^–7^ to 10^–3^*P*/*P*_0_, MIT-1 has a lower N_2_ uptake than MCM-22, which is consistent with the loss of the 10-ring channels associated with the 12-ring supercages along the *c*-axis.^26^ Taken together, the characterization data confirm that MIT-1 is a highly crystalline delaminated MWW material with high surface area and mesoporosity.

**Fig. 2:**
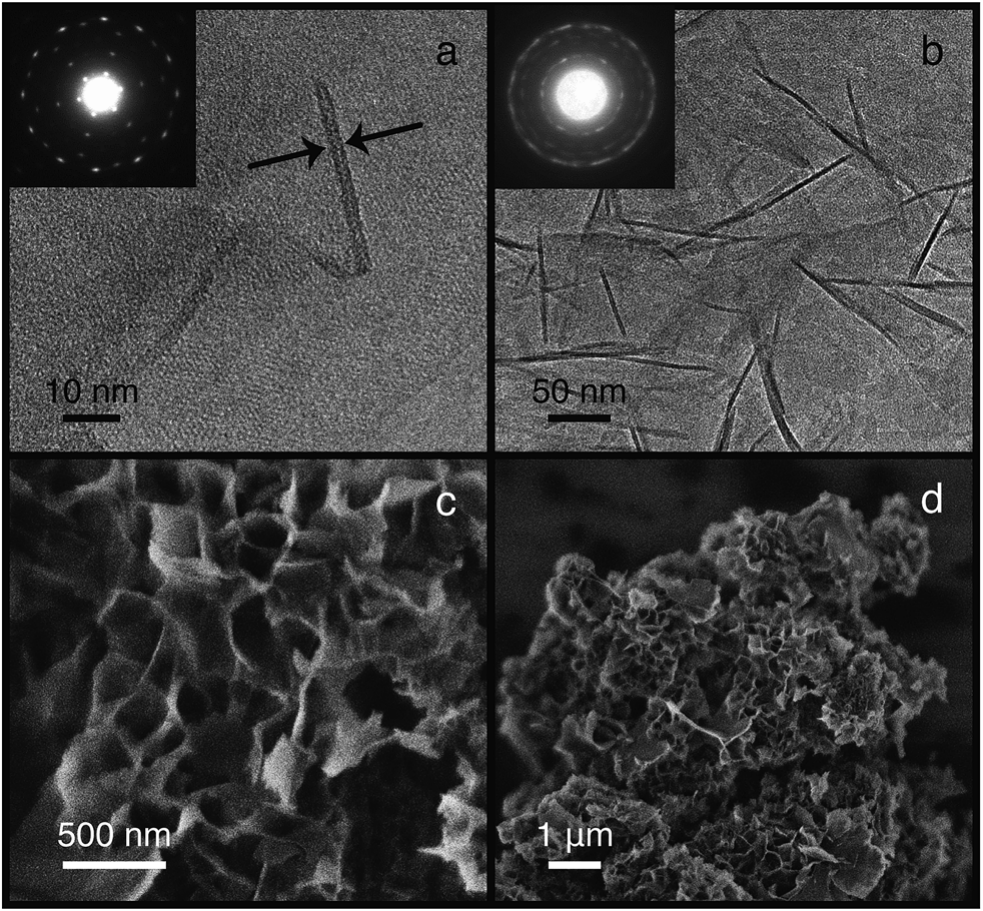
Transmission electron microscopy images of MIT-1 (a and b), with selected-area diffraction patterns perpendicular to the plane of sheets (inset). Scanning electron microscopy images of MIT-1 (c and d).

The coordination environment of Al atoms was analyzed by ^27^Al MAS NMR (see Fig. 1B). MIT-1 with a Si/Al_total_ = 16.2 (as quantified from elemental analysis) features mainly tetrahedrally-coordinated framework Al species at 55 ppm with only a small fraction (<8%) of octahedrally coordinated extra-framework Al species present at 0 ppm. Note that the amount of extra-framework Al increases to *ca.* 30% after calcination (see Fig. S11, ESI†), in agreement with previous reports by Corma *et al.* for MCM-22.^18^ Calcination conditions require further optimization to minimize dealumination.

The number of internal and external acid sites were investigated with ^31^P MAS NMR using trimethylphosphine oxide (TMPO) and tributylphosphine oxide (TBPO), respectively, as probe molecules. MCM-22, MCM-56, and MIT-1 have comparable peak signals at 85, 72, 68, and 63 ppm (Table S4 and Fig. S12, ESI†), which correspond to acid sites present in the 12-ring cages and 10-ring channels of MCM-22.^47^ These chemical shifts are associated with strong Brønsted acid sites as determined by theoretical calculations between proton affinities and ^31^P chemical shifts.^47^ Additional peaks at 53, 42, and 31 ppm correspond to TMPO adsorbed onto Lewis acidic extra-framework Al, physisorbed TMPO, and crystalline TMPO, respectively. The total number of acid sites were quantified using spectra integration coupled with elemental analysis, showing 46, 32, and 33 × 10^–5^ mol g^–1^ for MCM-22, MCM-56, and MIT-1, respectively (see Table S2, ESI†). Following the same procedure, the external acid sites were probed with TBPO (*ca.* 0.8 nm), which cannot fit inside 10-ring channels.^48^ MIT-1 had 21 × 10^–5^ mol g^–1^ of external acid sites, which is approximately three times more surface sites than those of MCM-22 (6 × 10^–5^ mol g^–1^) and two time more surface sites than those of MCM-56 (13 × 10^–5^ mol g^–1^). These values correspond well with the three and two-fold increases in external surface area for MIT-1 compared to MCM-22 and MCM-56.

A parametric study of Al, Na, and water content was conducted to determine the synthesis window for MIT-1. Table S5 and Fig. S13 (ESI†) show the resulting phases formed at different gel compositions. The synthesis space closely mirrors the space for phase-pure MCM-22(P). At Si/Al ratios below 12, only amorphous product is observed, while at Si/Al ratios above 70, competing MFI phases are observed. Materials synthesized in the absence of Al consistently resulted in a disordered MRE topology (ZSM-48).^49^–^51^ Increasing the NaOH/Si from 0.2 to 0.3 decreased the crystallization time from 14 to 7 days. Decreasing the NaOH/Si to 0.1 increased the crystallization time to 30 days. This modulation of crystallization time likely arises from the increase in OH^–^ content, since substituting NaCl as a sodium source resulted in an amorphous product. Increasing the H_2_O/Si ratio above 30 did not influence crystallization, but lower water contents generated only amorphous phases.

The Friedel–Crafts alkylation of benzyl alcohol (BA) with benzene was used as a model reaction to assess the catalytic activity of MIT-1. Both the *C*-alkylation (diphenylmethane (DP)) and *O*-alkylation (dibenzyl ether (DE)) products are unable to fit inside 10-ring pores, thus limiting catalytic activity to the external surface for regular 3D zeolites. As shown in Table 1, MIT-1 converts 49% of BA after 1.5 h at 358 K with a DP yield of 23%. Nearly full conversion is observed after 3 h reaction time with a 65% yield of DP. After 5 h, the yield of DP increases to 99% as DE is reversibly converted back to BA, which is *C*-alkylated into DP. In contrast, MCM-22 and MCM-56 only reach 40% and 44% conversion after 3 h, respectively, at comparable Al loadings. Thus, Al-normalized rates at similar conversion levels show that MIT-1 has a three-fold increase in activity. This reactivity profile is proportional to the increase in external surface area and external acid site concentration of MIT-1 compared to MCM-22. Bulk Al-MFI zeolites showed negligible activity due to their low external surface areas. Al-MCM-41 also showed low activity, in agreement with Na *et al.* who showed that strong Brønsted acid sites are required to catalyze this reaction.^4^

**Table 1:** Reactivity and selectivity data for the Friedel–Crafts alkyation of benzene with benzyl alcohol^*a*^

Catalyst	Conversion (%)	Yield DP (%)	Yield DE (%)
MCM-22	40	19	18
MCM-56	44	19	20
MIT-1	98	65	26
MIT-1^*b*^	49	23	21
MIT-1^*c*^	100	99	<1
Al-MCM-41	2	<1	<1
Al-MFI	3	<1	2

^*a*^Reaction conditions: BA/Al = 200 mol mol^–1^, 6.5 wt% BA in benzene, 3 h, 358 K.

^*b*^1.5 h.

^*c*^BA/Al = 100 mol mol^–1^, 5 h.

## Conclusions

We present the first one-pot synthesis of MWW zeolite nanosheets with high surface area and high crystallinity. Rational design of OSDAs can be generalized for the synthesis of other zeolite topologies with open architectures, which are needed to address new challenges arising from our increasing need to convert bulky substrates. Current efforts in our group are focused on investigating other OSDA designs.

## Supplementary Material

Supplementary information
