# Supplementary material for: One-pot synthesis of MWW zeolite nanosheets using a rationally designed organic structure-directing agent
Source: Chem Sci. 2015 Jul 22;6(11):6320–4. doi: 10.1039/c5sc01912e (PMC4603534; doi:10.1039/c5sc01912e)
Supplement: Supplementary file 1 [file SC-006-C5SC01912E-s001.pdf]

Electronic Supplementary Material (ESI) for Chemical Science.
This journal is © The Royal Society of Chemistry 2015

Supporting Information

One-pot synthesis of MWW zeolite nanosheets using a rationally designed organic structure-directing agent

Helen Y. Luo[†], Vladimir K. Michaelis[‡], Sydney Hodges[†], Robert G. Griffin[‡], Yuriy Román-Leshkov*[,†]

[†]Department of Chemical Engineering and [‡]Department of Chemistry and Francis Bitter Magnet Laboratory, Massachusetts Institute of Technology, Cambridge, Massachusetts 02139, United States

*Email: yroman@mit.edu

1. Experimental Methods

1.1 Synthesis of Ada-(i=4,5,6)-16 OSDA

Ada-(4,5,6)-16 OSDA was synthesized in three steps. First, 1-adamantylamine (97 %, Sigma Aldrich) was dissolved in a 3-fold molar excess of formaldehyde in aqueous solution (37%, Sigma Aldrich) and heated to 373 K under refluxing. A 3-fold molar excess of formic acid (95%, Sigma Aldrich) was added slowly to the mixture over the span of 2 h, and refluxed for another 3 h. The solution was removed from heat, and neutralized to pH = 14 with sodium hydroxide solution (8 M, Sigma Aldrich). The product, dimethyl-1-adamantylamine (Ada-N(Me)$_2$), was liquid-liquid extracted from the mixture with diethyl ether and dried with potassium carbonate (99 wt%, Sigma Aldrich). The diethyl ether was removed by rotary evaporation. Ada-N(Me)$_2$ structure was verified by ^{1}H nuclear magnetic resonance (NMR) (Figure S4). Second, ~2 g of Ada-N(Me)$_2$, and a 10-fold molar excess of 1,4-dibromobutane (1,5-dibromopentane or 1,6–dibromohexane) (99%, Sigma Aldrich) were dissolved in 50 ml of acetonitrile (anhydrous 99.8%, Sigma Aldrich), and refluxed at 355 K for 16 h. The solvent was removed by rotary evaporation, and the product (Ada-N$^+$(Me)$_2$-(4,5,6)-Br,Br$^-$) was recrystallized from dichloromethane and washed with diethyl ether. Ada-N$^+$(Me)$_2$-(4,5,6)-Br,Br$^-$ structure was verified by ^{1}H NMR (Figure S5). Third, ~2 g of Ada-N$^+$(Me)$_2$-(4,5,6)-Br,Br$^-$ and a 3-fold molar excess of N,N-dimethylhexadecylamine (98%, TCI Chemicals) were dissolved in 50 ml of acetonitrile, and refluxed at 355 K for 16 h. The solvent was removed by rotary evaporation, and the product, Ada-(4,5,6)-16 in bromide form, was washed with diethyl ether. Ada-(4,5,6)-16 structure was verified using ^{1}H and ^{13}C NMR (Figure S6). The product was converted from the bromide to hydroxide form using hydroxide exchange resin (Amberlite IRN-78 hydroxide form, Supelco) in water. The product was titrated using HCl.

1.2 Synthesis of MIT-1

MIT-1 was synthesized by combining the aqueous solution of Ada-4-16 in hydroxide form with sodium hydroxide (99.99% trace metals basis, Sigma Aldrich), and water in a Teflon jar. The mixture was stirred for 15 min, and then aluminum hydroxide (80.3% $Al(OH)_3$, SPI Pharma 0250 aluminum hydroxide powder) was added. The mixture was stirred for 15 min, and colloidal silica (LUDOX® LS 30) was added. The mixture was allowed to age under stirring for 4 h at room temperature. For some samples, water evaporation was necessary and was facilitated by a stream of air and measured gravimetrically. The final gel composition was 1 SiO_2/0.1 OSDA/0.05 $Al(OH)_3$/0.2 NaOH/45 H_2O. The gel was transferred to a Teflon-lined Parr reactor, sealed, and held at 433 K at 60 rpm rotation. Aliquots of the synthesis mixture were taken periodically and monitored by powder X-ray diffraction (PXRD). After crystallization, the solids were recovered by filtration, extensively washed with deionized (DI) H_2O, and dried at 373 K overnight. The solid was calcined by heating under flowing N_2 at a rate of 2 K/min with a 1 h hold at 423 K, a 2 h hold at 573 K, and a 3 h hold at 813 K. The flowing gas was then switched to dry air and the temperature was held at 813 K for another 6 h.

1.3 Synthesis of MCM-22 and MCM-56

MCM-22 is synthesized following the methods of Corma et al.[1] First, 0.27 g of NaOH, 0.16 g of sodium aluminate (53% Al_2O_3, 45% Na_2O, 2% H_2O, Sigma Aldrich), are dissolved in 40 g of DI H_2O. Next, 3.0 g of

fumed silica (Cabosil M5) are slowly added to the solution, and 1.8 g of hexamethyleneimine (HMI) are added dropwise. The mixture is aged under stirring at room temperature for 1 h. The final gel composition was 1 SiO_2/0.35 HMI/0.033 Al/0.18 Na/45 H_2O. The gel was transferred to a Teflon-lined Parr reactor, sealed, and held at 423 K at 60 rpm rotation for 11 d. MCM-56 is synthesized following the methods of Fung et al.[2] Using the same precursors, with the exception of using the colloidal silica solution Ludox LS30 instead of fumed silica, the final gel composition was 1 SiO_2/0.35 HMI/0.083 Al/0.21 Na/19.9 H_2O. The gel was transferred to a Teflon-lined Parr reactor, sealed, and held at 418 K at 60 rpm rotation with a Teflon stir-bar added to improve mixing for 33 h. After crystallization, the solids were recovered by filtration, extensively washed with deionized (DI) H_2O, and dried at 373 K overnight. The solids were calcined by heating under flowing dry air at a rate of 2 K/min with a 1 h hold at 423 K, a 2 h hold at 573 K, and a 6 h hold at 823 K.

1.4 Characterization

PXRD patterns were collected using a Bruker D8 diffractometer using Cu Kα radiation (40 kV, 40mA). Nitrogen isotherms were measured on a Quantachrome Autosorb iQ at liquid nitrogen temperature (77.35 K). The external surface areas were calculated by the *t*-plot method, and micropore and total pore volumes were determined at P/P_0=0.01 and 0.95, respectively. Pore volumes were calculated using the Non-Local Density Function Theory (NLDFT) method for N_2 on silica on the adsorption branch at 77 K with a cylindrical pore model (ASiQwin, Quantachrome Instruments). Scanning electron microscopy (SEM) images were acquired on a JEOL 6700F at an accelerating voltage of 1 kV. Transmission electron microscopy (TEM) images and selected-area diffraction patterns were acquired on a JEOL 2010F at an operating voltage of 200 kV. Elemental analysis was performed with a CCD-based inductively coupled plasma (ICP) atomic emission spectrometer (Activa-S, HORIBA Scientific). Samples were dissolved in 48% HF and diluted into 3% HNO3 before analysis. A 5-point calibration curve was built using the following ICP standards: 1000 ppm Al in 3% HNO_3, 1000 ppm P in H_2O (all TraceCERT®) on the 308.215 nm Al line and the 177.440 nm P line. Simulated PXRD patterns were generated using powder pattern theorem implemented with UDSKIP.[3] Atomic coordinates for zeolite MWW structure were obtained from Camblor et al.[4] The simulated crystal had dimensions of 15 unit cells in the *a*- and *b*-directions, and 1 unit cell in the *c*-direction.

Magic-angle spinning (MAS) NMR was acquired using home-built 700 and 360 MHz spectrometers (courtesy of Dr. D. Ruben, FBML-MIT) equipped with 3.2 and 4 mm Varian-Chemagnetics triple resonance H/X/Y MAS probes. ^{27}Al MAS NMR (700 MHz, ^{1}H) spectra were obtained from fully hydrated zeolite samples using a short quantitative[5] single (Bloch) pulse (9° flip-angle) at a spinning frequency of 14 KHz, recycle delay of 2 s and between 4-8 k scans. An aqueous 1.0 M solution of $Al(NO_3)_3$ was used as an external reference (0 ppm). Trimethylphosphine oxide (TMPO, 99% Alfa Aesar) and tributylphosphine oxide (TBPO, 97% Alfa Aesar) were added to samples through vapor phase deposition. First samples were degassed overnight under dynamic vacuum at 423 K in a glass tube with a stopcock. Solid TMPO or TBPO was added to the sample under inert N_2 atmosphere in a glovebox. The glass tube was then evacuated at room temperature, isolated from vacuum, and allowed to equilibrate at 373 K for 3 h. The samples were cooled and packed under inert atmosphere into MAS rotors with gastight caps. ^{31}P MAS NMR (360 MHz, ^{1}H) spectra were collected using a Bloch experiment with a pulse width of 3.5 μs ($\gamma B_1/2\pi$

= 71 kHz), an optimized recycle delay of 5 s, and a spinning frequency of 10 kHz. ^{31}P chemical shifts were referenced to an aqueous solution of 85% H_3PO_4 (0 ppm). A Lorentzian deconvolution method was used to analyze the ^{31}P MAS NMR spectra. Quantitative analysis of acid site concentrations were calculated using a method described by Zhao et al.[6] Total acid site concentration was calculated by: (mol P from ICP)*(TMPO chemisorbed / TMPO total from NMR) / g catalyst. Total external acid site concentration was calculated by: (mol P from ICP)*(TBPO chemisorbed / TBPO total from N MR) / g catalyst. Specific peak areas are available in Table S5. ^{13}C MAS NMR (700 MHz, ^{1}H) were acquired using a cross-polarization[7] experiment with a 1.5 ms contact time and a ramp on ^{13}C (^{1}H $\gamma B_1/2\pi$ = 50 kHz and ^{13}C $\gamma B_1/2\pi$ = 65 kHz). All data were acquired using a spinning frequency of 12.5 kHz, variable recycle delays (i.e., 1.3xT_1, optimized from the ^{1}H MAS NMR Bloch spectra, 256 scans, 5µs pulse (^{1}H $\gamma B_1/2\pi$ = 50 kHz) and between 4,096 and 16,384 co-added transients depending on the signal-to-noise. Two-dimensional ^{13}C-{^{1}H} HETCOR spectra were acquired under identical conditions above, 1,024 co-added transients and 100 increments (40 µs dwell) in the indirect dimension. All ^{13}C data were acquired with high power two-pulse phase modulation (TPPM)[8] proton decoupling (^{1}H $\gamma B_1/2\pi$ = 83 kHz). ^{13}C spectra were referenced to adamantane (40.49 ppm) with respect to DDS (0 ppm).

1.5 Friedel-Crafts alkylation reactions

Prior to reaction, all materials were ion exchanged with a 0.2 M ammonium acetate solution ($NH_4C_2H_3O_2$/Al=10) for 16 h and filtered with DI H_2O. The exchange is done three separate times. The solids are then dried and calcined using the methods described in section 1.2 and 1.3. The alkylation of benzene with benzyl alcohol reactions were performed in septum-sealed, thick-walled glass reactors with magnetic stirring. Typically, 5 g of 6.5 wt% benzyl alcohol (BA) in benzene and ~30 mg catalyst (specific amount of catalyst adjusted such that mol BA/mol Al = 200) were sealed into the vial and placed in a silicon oil bath at 358 K. Samples were taken at 1.5 h and 3 h by cooling the vial to room temperature, taking an aliquot, and filtering the solid. A known amount of 1,3,5-tri-tert-butylbenzene was added as an external standard. The solution was analyzed on a GC-FID (Agilent 6890N) using an HP-1 capillary column (J&W Scientific/Agilent Technologies; 30m long, 0.25mm i.d., 0.25 µm thick). The conversion was calculated based on the mole of BA reacted compared to the mole of BA in the feed; the yield is calculated as the mole of diphenylmethane (DP) or dibenzyl ether (DE) normalized by the mole of BA in the feed. Al-MCM-41 and Al-MFI were purchased from ACS Materials with product codes: Al-MCM-41, and ZSM-5 (P-38).

1.6 Molecular Modeling

The location and van der Waals (vdW) interaction energy of the Ada-4-16 OSDA with the MWW zeolite nanosheet were studied by molecular mechanics simulations using the Materials Studio 6.1 software[9]. The CVFF forcefield[10] was selected for the calculation as implemented by the Discover Module. A cutoff of 12.5 Å was employed for vdW term of the atom-atom interactions. Ewald summation was employed to compute the electrostatic energies of the system. The simulation cell consisted of 9 unit cells of MWW (a x b x c = 3 x 3 x 1). A vacuum of 30 Å was added perpendicular to the surface in the c-direction. The oxygen atoms at the zeolite surface were protonated. The positive charge of the system due to the quaternary ammoniums in the OSDA was compensated with a uniform charge background. The most

stable locations for the OSDA molecule were obtained by simulated annealing. The Ada-4-16 was placed on the surface of the zeolite in a variety of configurations with the C16 tail oriented away from the surface. Molecular dynamics (MD) simulations were performed at 343 K for 10 ps using the *NVE* ensemble. The geometry of the equilibrated system was optimized to find the lowest energy conformations.

2. Tables

Table S1. Synthesis of MWWns varying OSDA, temperature, and co-template.

Entry	OSDA i	OSDA/Si	Co-template Ada-OH/Si	Temperature (K)	Time (days)	Observed phase
1	4	0.1	0	433	14	MIT-1
2	4	0.1	0	423	31	MIT-1
3	4	0.2	0	433	13	MIT-1
4	4	0.3	0	433	13	MIT-1
5	5	0.1	0	433	30	Amorphous
6	6	0.1	0	433	22	MIT-1
7	6	0.1	0	423	31	MIT-1/amorphous
8	4	0.1	0.00625	433	18	MIT-1
9	4	0.1	0.0125	433	18	MIT-1
10	4	0.1	0.025	433	8	CHA
11	4	0.1	0.05	433	5	CHA
12	4	0.1	0.1	433	5	CHA

Table S2. Properties of MCM-22, MCM-56, and MIT-1 zeolites.

Catalyst	Si/Al[a]	Micropore volume[b] (cm^3g^{-1})	Total pore volume[c] (cm^3g^{-1})	S_{ext}[d] (m^2g^{-1})	Total acid sites[e] (x 10^{-5} mol g^{-1})	External acid sites[f] (x 10^{-5} mol g^{-1})
MCM-22	25	0.142	0.289	121	46	6
MCM-56	12	0.133	0.601	219	32	13
MIT-1	16	0.131	1.014	513	33	21

a. Si/Al determined by ICP-AES elemental analysis, b. micropore volume calculated from N_2 adsorption isotherm at P/P_0=0.01, c. total pore volume calculated from N_2 adsorption isotherm at P/P_0=0.95, d. external surface area S_{ext} calculated using the t-plot method, e. total acid sites calculated from TMPO titration and ^{31}P MAS NMR, f. external acid sites calculated from TBPO titration and ^{31}P MAS NMR.

Table S3. Theoretical calculations for MWW zeolite nanosheet surface areas.

Entry	a (nm)	b (nm)	c (nm)	Calculated external surface area (m^2g^{-1})
1	150	150	2.5	517
2	100	100	2.5	527
3	80	80	2.5	532
4	150	150	5	267
5	100	100	5	275
6	80	80	5	282

Theoretical surface area per unit mass of MWW nanosheets were calculated using geometric arguments. For MWW, the following cell dimensions (obtained from the International Zeolite Association website)[11] a=14.390, b=14.390, c=25.198 Å. Volume of the cell = 4518.84 $Å^3$ with a density of 0.0159 atom/$Å^3$.

Table S4. TMPO and TBPO fractional peak areas from ^{31}P MAS NMR spectra.

TMPO peak	85 ppm	72 ppm	68 ppm	63 ppm	53 ppm	42 ppm	31 ppm
MCM-22	0.01	0.17	0.25	0.03	0.09	0.36	0.08
MCM-56	<0.01	0.08	0.10	0.14	0.11	0.57	<0.01
MIT-1	<0.01	0.08	0.13	0.08	0.04	0.66	0.01
TBPO peak	91 ppm	-	75 ppm	73 ppm	57 ppm	52 ppm	46 ppm
MCM-22	<0.01	-	0.07	0.25	-	0.68	<0.01
MCM-56	<0.01	-	0.27	0.17	0.11	0.45	<0.01
MIT-1	<0.01	-	0.34	0.05	-	0.43	0.18

Peak deconvolutions shown in Figure S12.

Table S5. Parametric study of the synthesis conditions of Al, Na, H_2O content screen.

Screen	Gel Si/Al	Product Si/Al[a]	Gel Na/Si	Gel H_2O/Si	Time (days)	Observed phase
1	21	-	0.00	44	30+	amorphous
2	20	22	0.10	45	30	MIT-1
3	21	19	0.20	44	14	MIT-1
4	19	16	0.31	46	7	MIT-1
5	20	-	0.20	10	21	MWW/amorphous
6	20	-	0.21	20	30+	amorphous
7	19	19	0.23	100	14	MIT-1
8	10	-	0.20	44	30+	amorphous
9	27	26	0.21	44	14	MIT-1
10	49	45	0.20	45	9	MIT-1
11	0	0	0.20	44	4	ZSM-48
12	10	-	0.20	10	30+	amorphous
13	10	-	0.20	20	30+	amorphous
14	10	-	0.20	92	30+	amorphous
15	30	-	0.20	20	23	MFI/amorphous
16	29	27	0.21	99	14	MIT-1
17	19	21	0.22	29	23	MIT-1
18	12	-	0.21	29	30+	amorphous
19	0	-	0.20	11	12	ZSM-48
20	0	-	0.20	29	5	ZSM-48
21	0	-	0.22	90	4	ZSM-48
22	99	-	0.20	44	4	MFI/amorphous
23	15	15	0.25	45	12	MIT-1
24	10	-	0.40	45	7	MOR
25	70	67	0.20	44	7	MIT-1
26	75	-	0.20	45	7	MWW/MOR
27	19	-	0.21 NaCl	46	30+	amorphous
28	20	-	0.20 KOH	45	14	MIT-1/amorphous
29	15	-	0.21	45	21	MIT-1/amorphous
30	12	13	0.30	45	14	MIT-1

a. Si/Al determined by ICP-AES elemental analysis

3. Figures

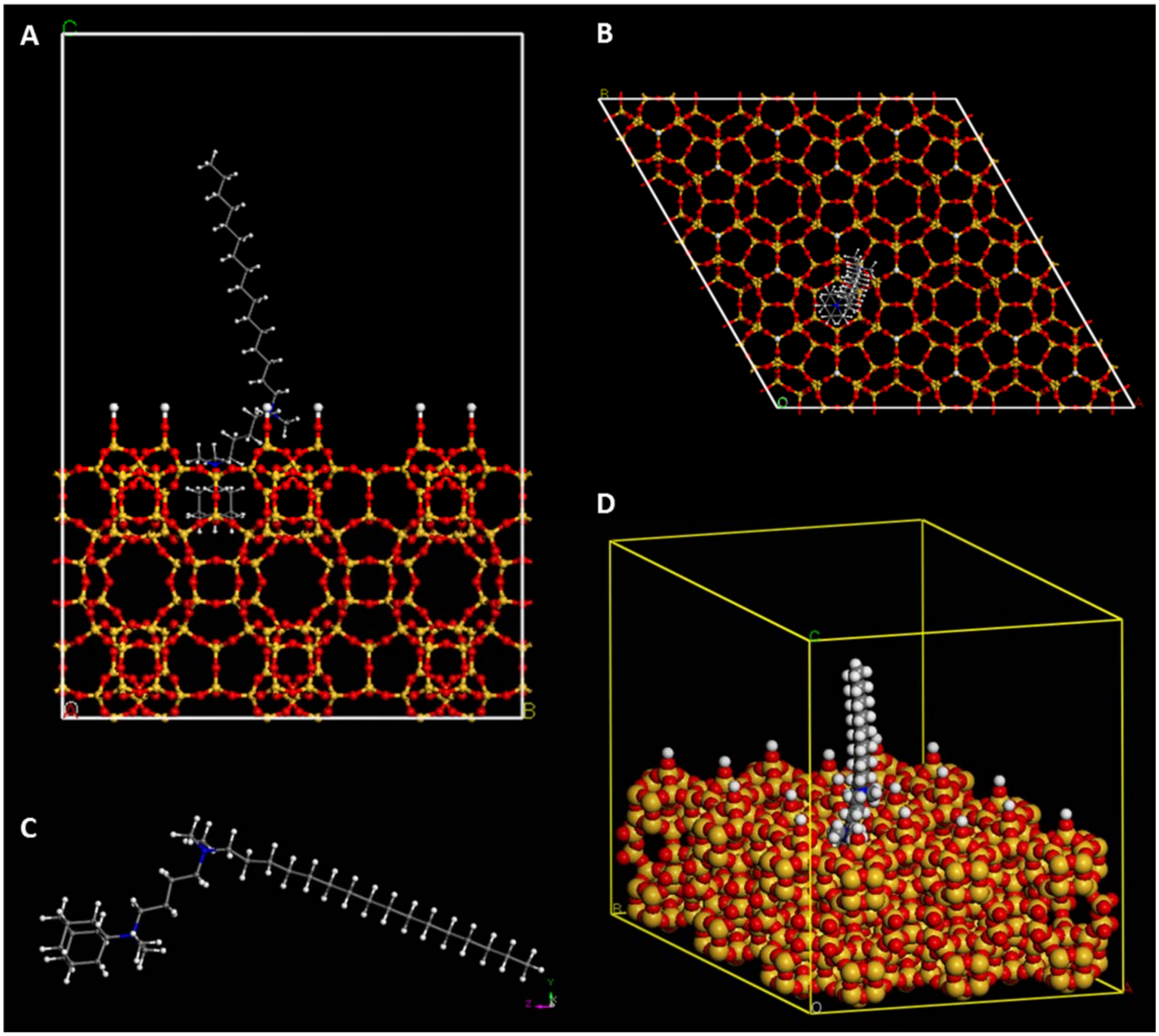

Figure S1. Optimized geometry after simulated annealing to sample the lowest energy conformations of the Ada-4-16 OSDA on the surface of MWW zeolite. Ada-4-16 (C) sits in the cups of the MWW framework (A, B, D) with the quaternary ammoniums stabilizing the cup openings. The tail is labile and moves freely during the simulated annealing.

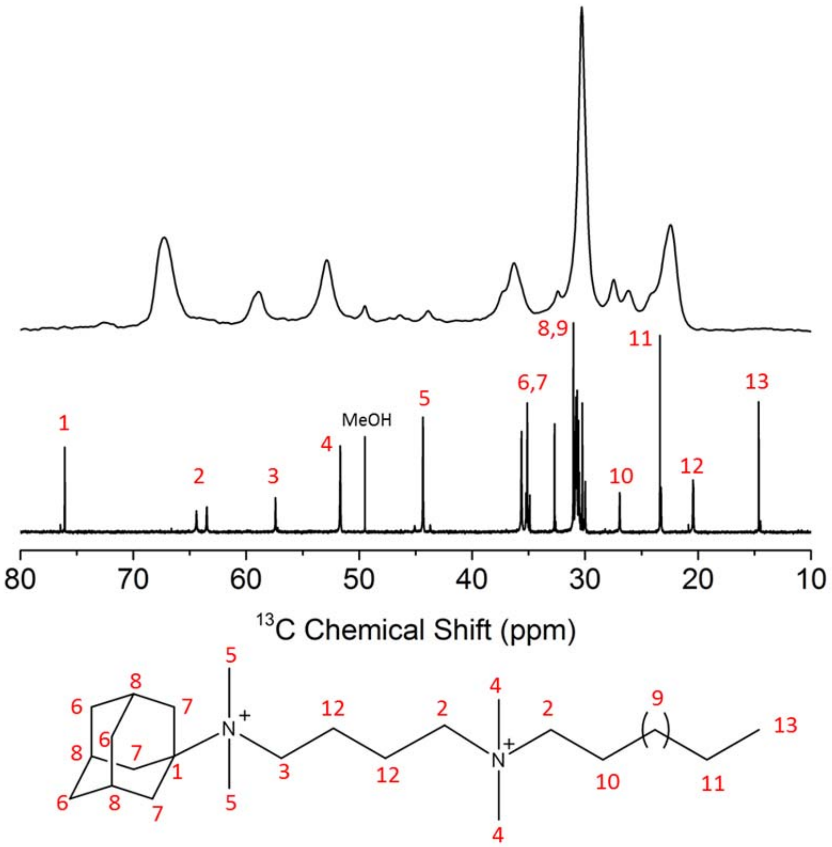

Figure S2. ^{13}C NMR of Ada-4-16 in D_2O with methanol reference (bottom). ^{13}C MAS NMR of Ada-4-16 occluded in MIT-1 pores.

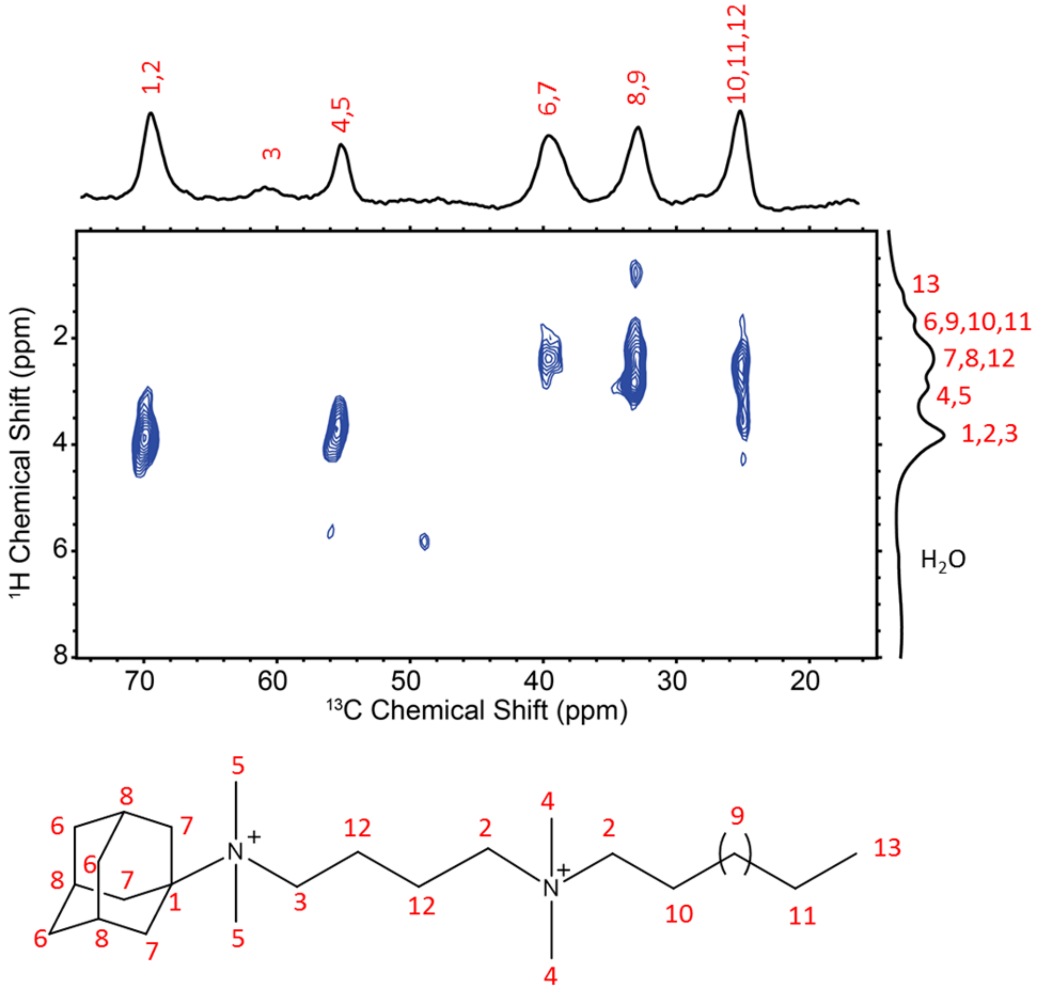

Figure S3. 2D ^{13}C-^{1}H HETCOR MAS NMR spectrum of Ada-4-16 occluded in MIT-1 pores. Tentative peak assignments are based on the 1D liquid phase ^{13}C and ^{1}H spectra of Ada-4-16 (Figure S2, S6).

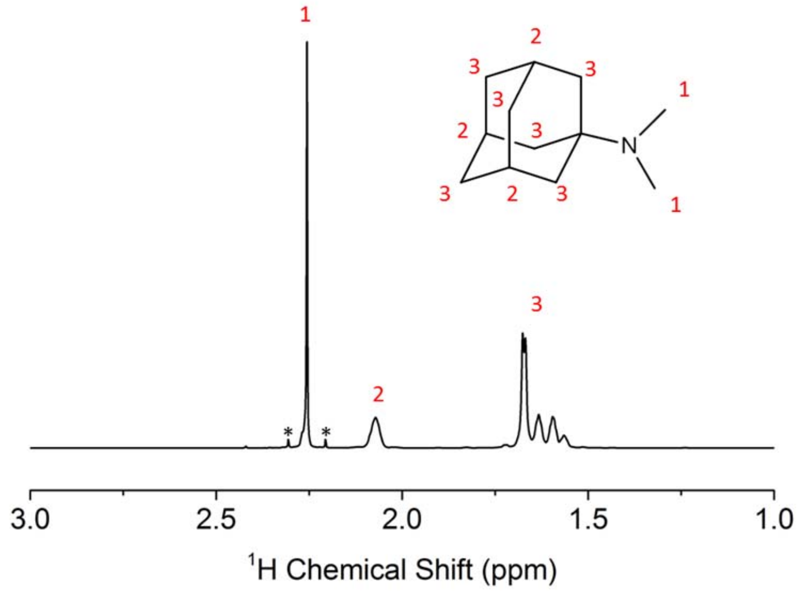

Figure S4. ^{1}H NMR of Ada-N(Me_2) in $CDCl_3$.

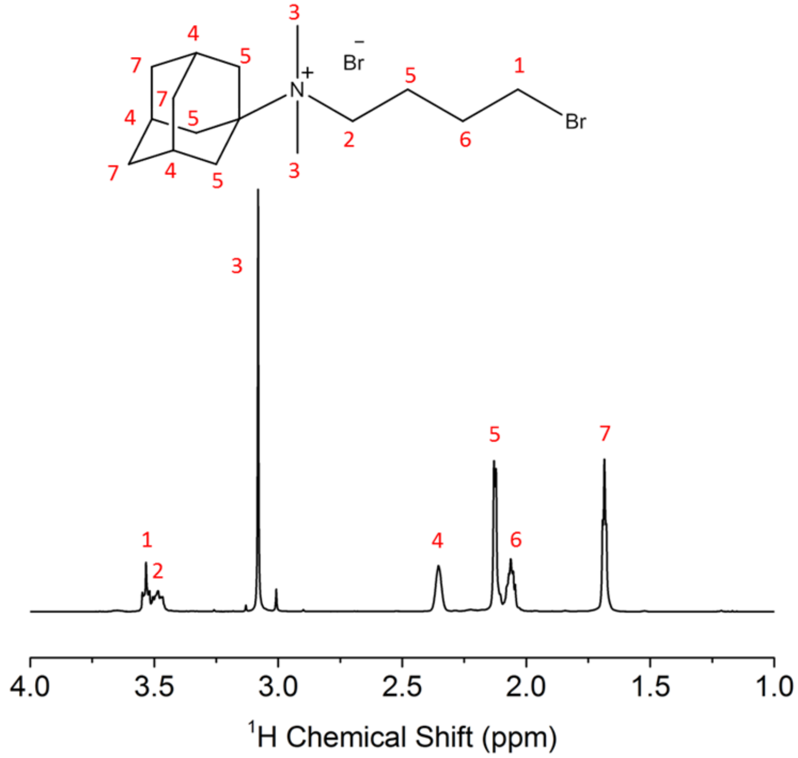

Figure S5. ^{1}H NMR of Ada-$N^+(Me)_2$-4-Br,Br^- in $CDCl_3$.

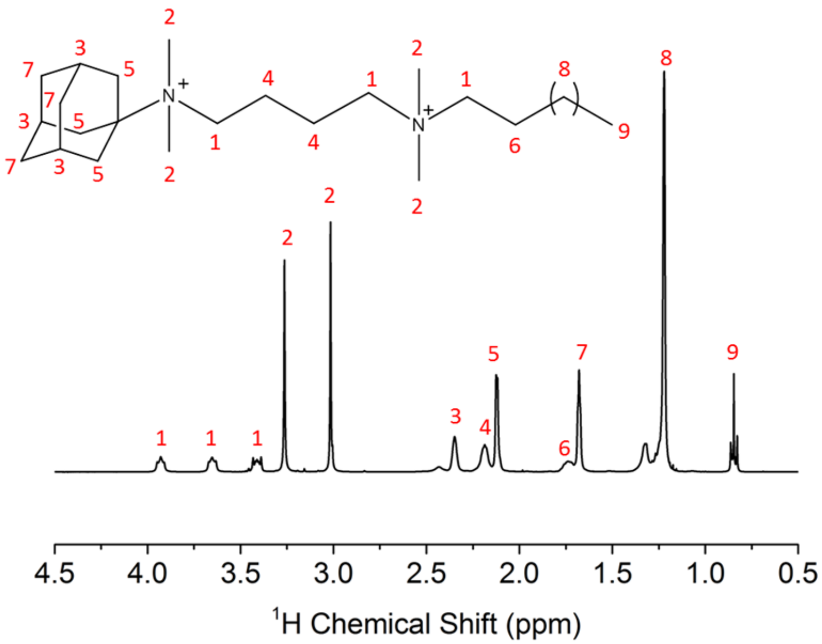

Figure S6. ^{1}H NMR of Ada-4-16, Br form in $CDCl_3$.

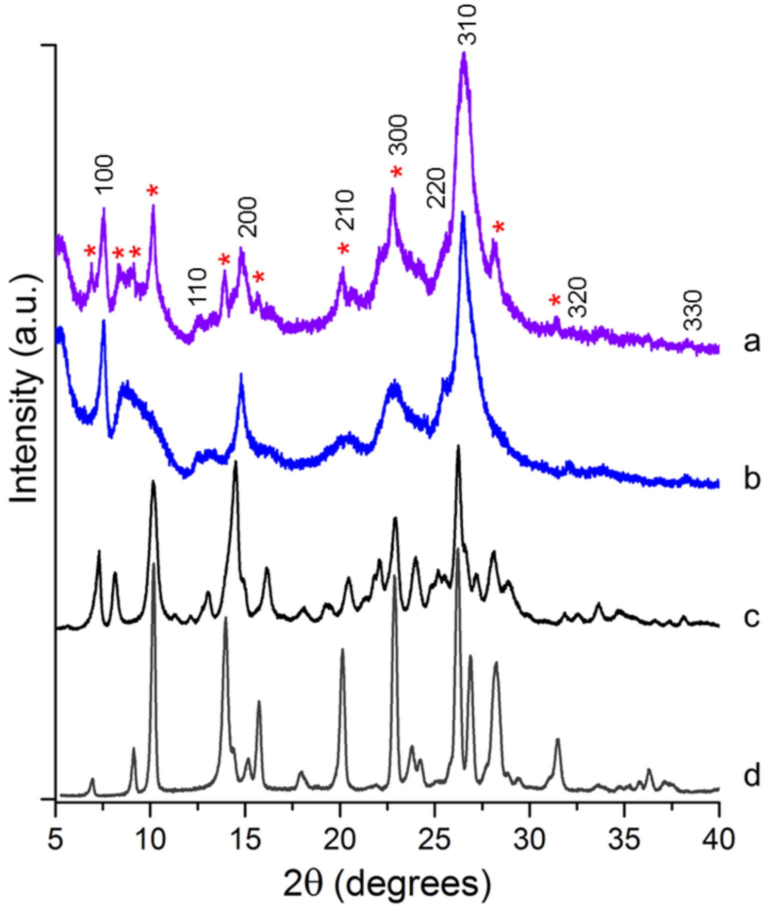

Figure S7. Powder XRD pattern of calcined mordenite (d), MCM-22 (c), MIT-1, 14 days (b), and MIT-1, 21 days (a). Red asterisks indicate growth of competing MOR phases and/or possible condensation of nanosheets into 3D MWW.

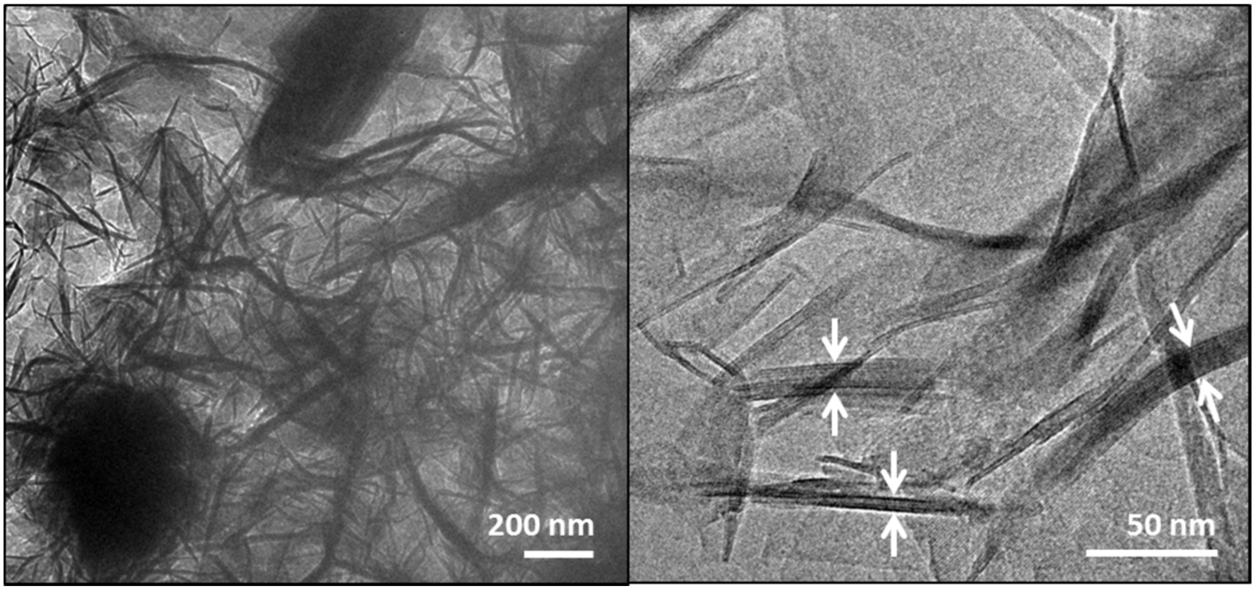

Figure S8. TEM images of calcined MIT-1 after 21 days of crystallization. Crystals around 500 nm in size of possible MOR impurities can be seen in low magnification images (left). Condensed sheets that form multilamellar structures that are 5-10 unit-cell layers (10-30 nm in thickness) thick can be seen in higher magnification images (right). These condensed layers are ubiquitous in the sample as seen in the low magnification image.

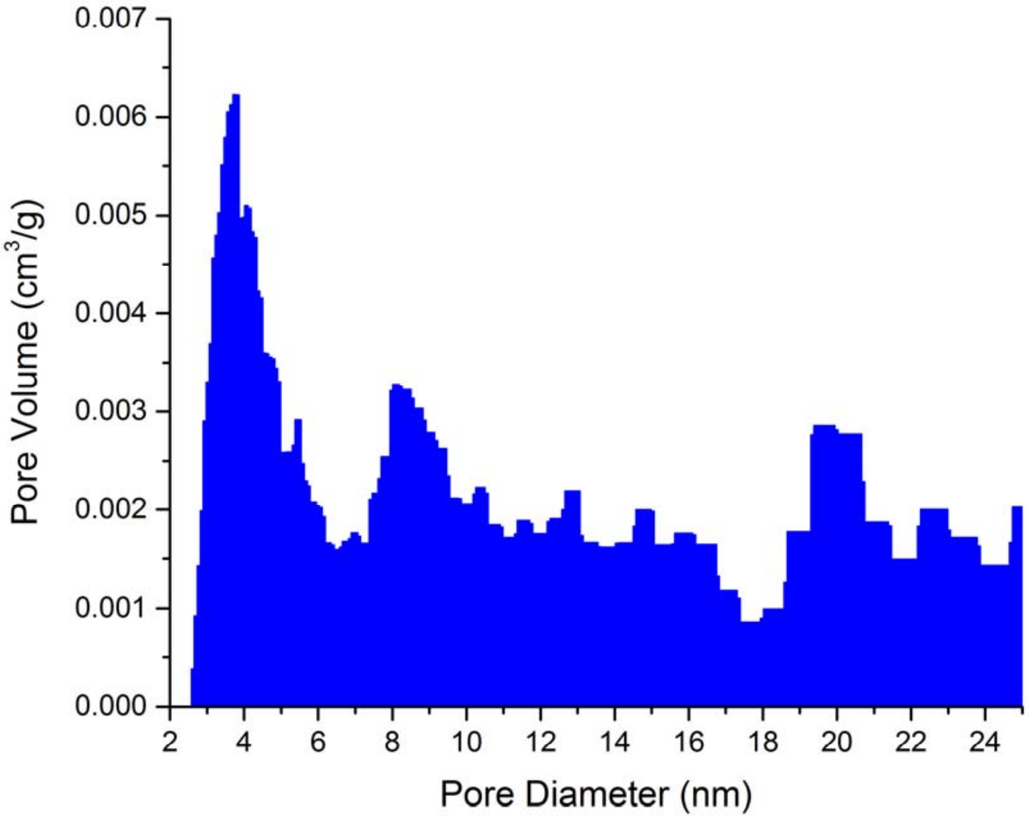

Figure S9. Pore volume histogram of MIT-1 using the Non-Local Density Function Theory (NLDFT) method for N_2 on silica on the adsorption branch at 77 K with a cylindrical pore model (ASiQwin, Quantachrome Instruments).

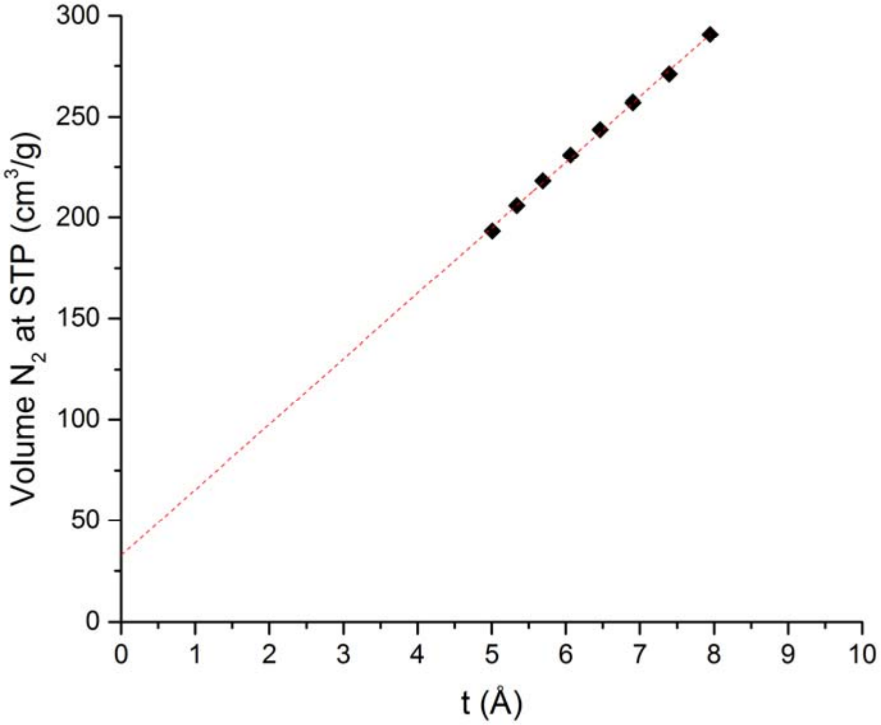

Figure S10. de Boer *t*-plot micropore analysis for MIT-1.

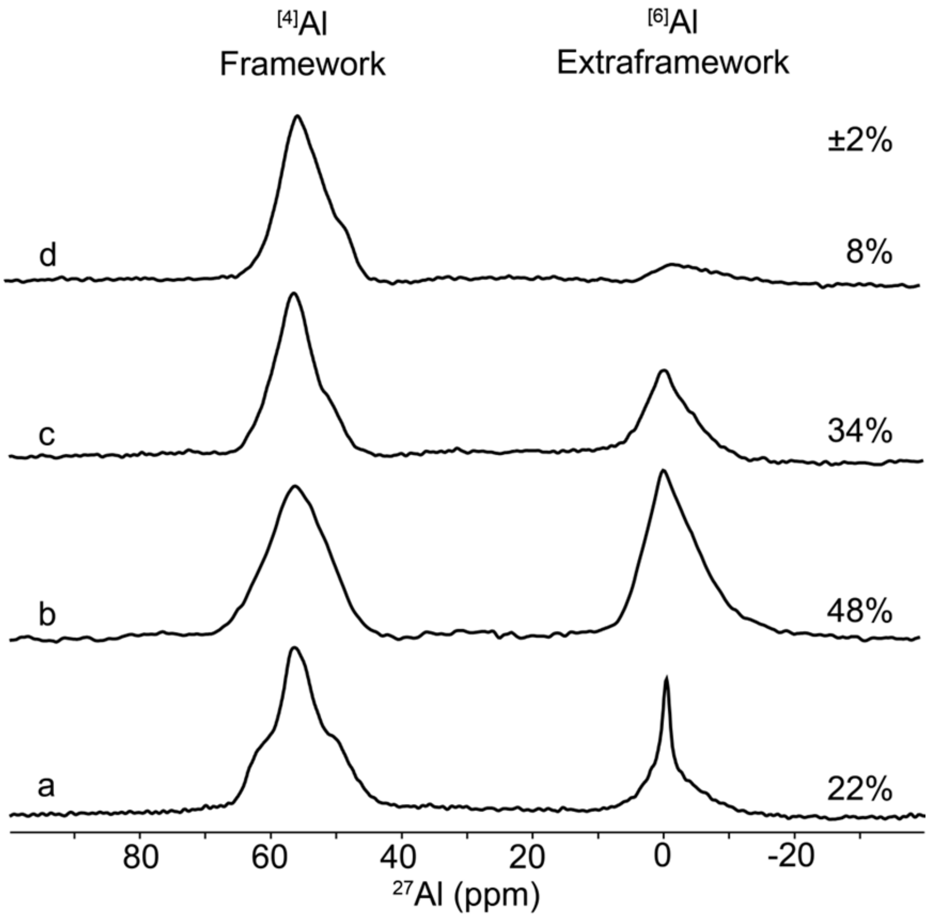

Figure S11. ^{27}Al MAS NMR spectra of calcined MCM-22 (a), MCM-56 (b), MIT-1 (c), and as-synthesized MIT-1 (d). Percentage of extraframework Al is labeled on the right above each spectrum.

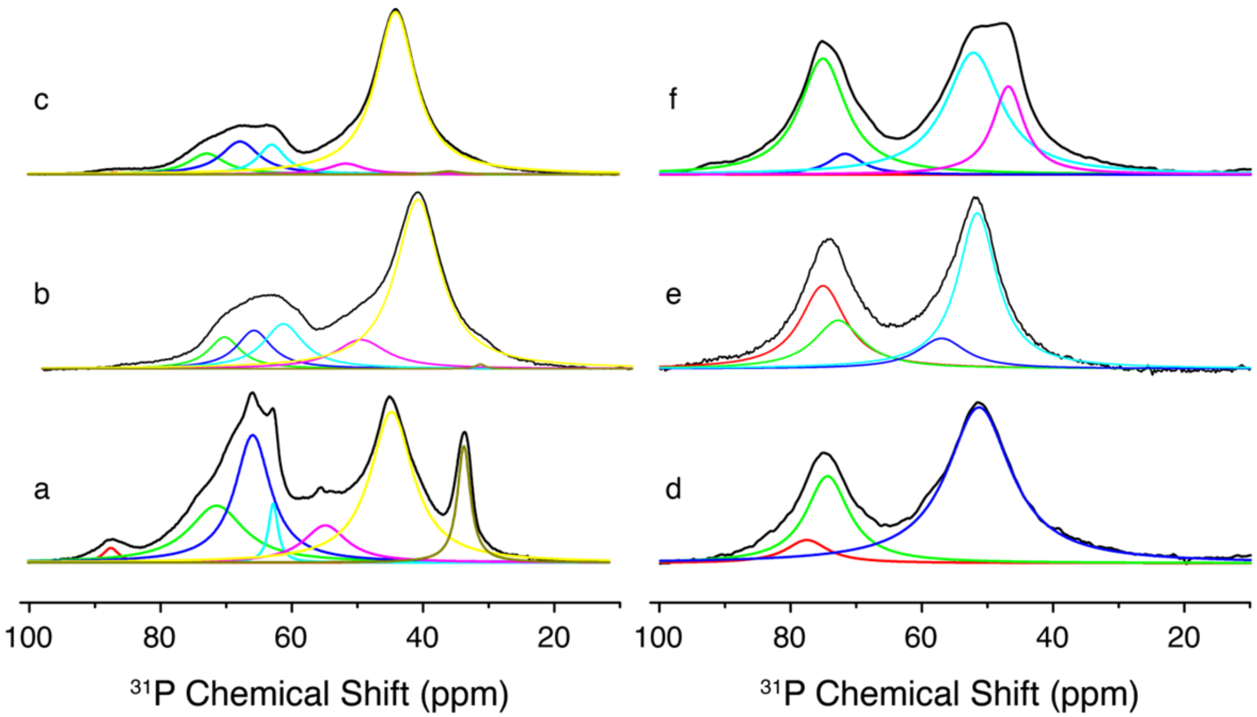

Figure S12. ^{31}P MAS NMR spectra of TMPO adsorbed onto MCM-22 (a), MCM-56 (b), MIT-1 (c); and TBPO adsorbed onto MCM-22 (d), MCM-56 (e), MIT-1 (f). A Lorentzian method was used for peak deconvolution.

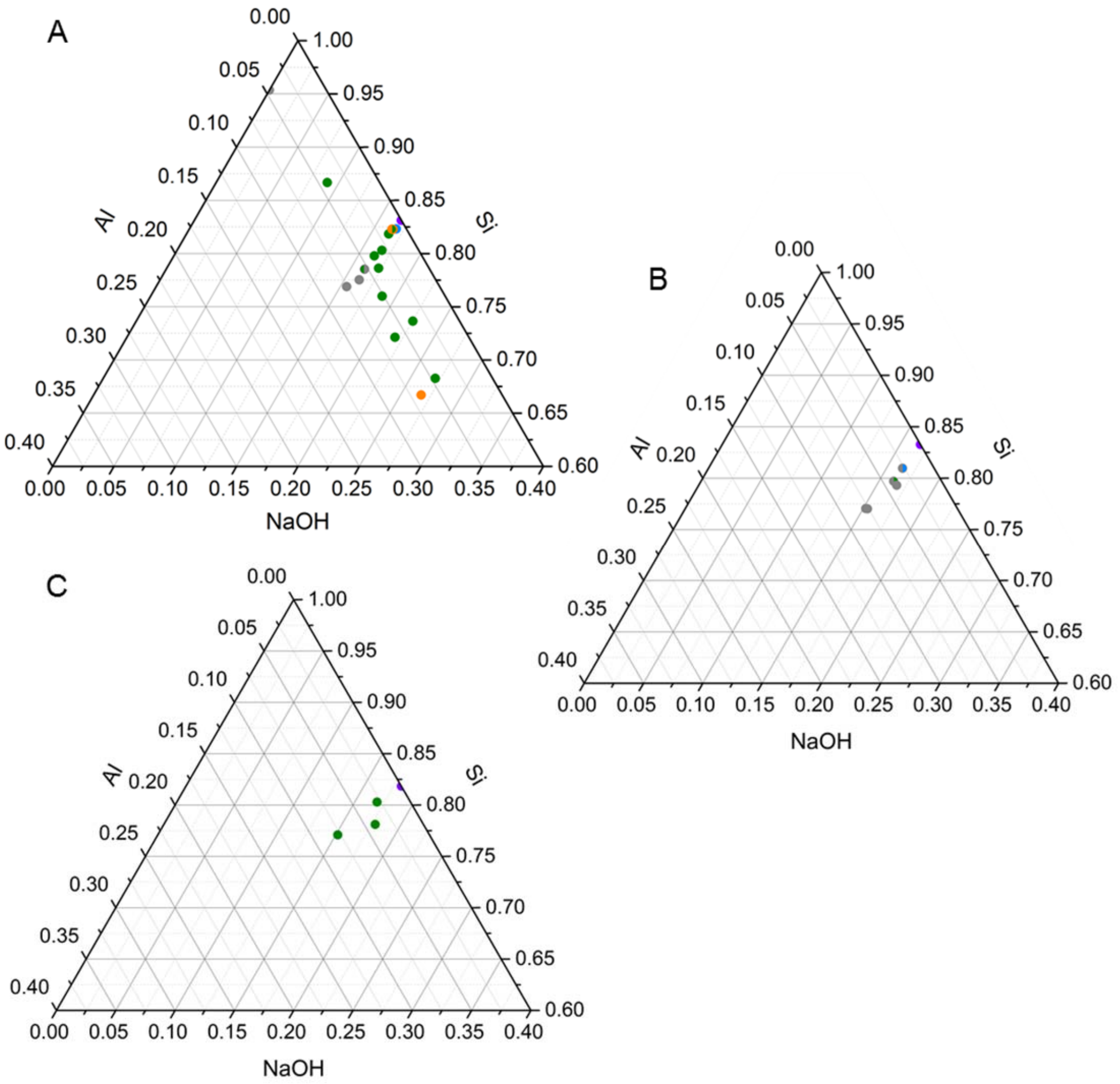

Figure S13. Ternary diagrams of synthesis gel compositions (Na, Al, Si) at H_2O/Si = 30,45 (A), H_2O/Si = 10,20 (B), H_2O/Si = 100 (C). Phase key: MIT-1 (green), amorphous (gray), MFI (blue), MOR (orange), MRE (purple).

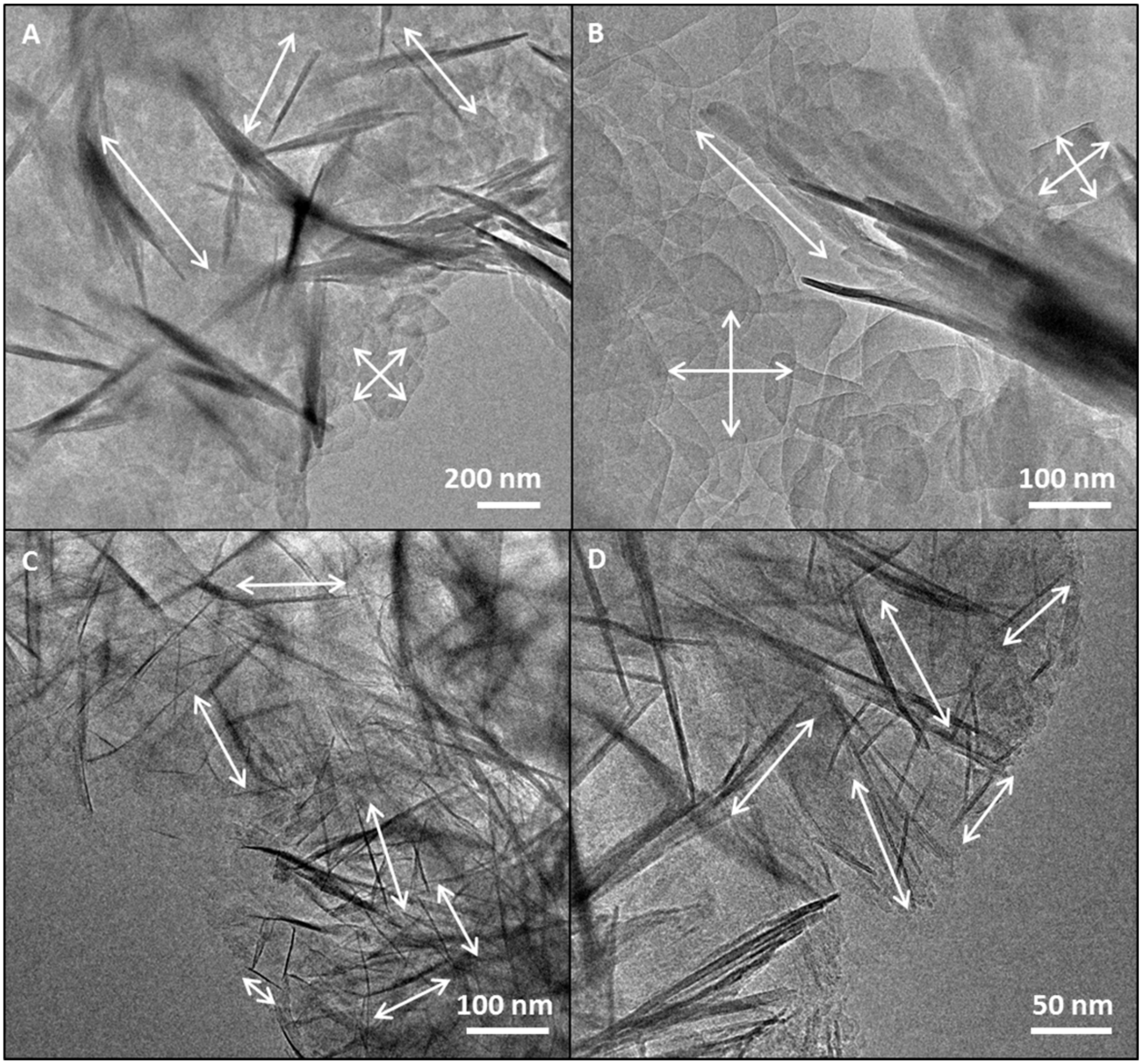

Figure S14. TEM images of calcined MCM-22 (A,B) and MCM-56 (C,D). Crystal sizes for MCM-22 span the range from 50-500 nm in the *a*- and *b*-directions and 10-50 nm in the *c*-direction. Crystals sizes for MCM-56 span the range from 50-200 nm in the *a*- and *b*-directions and 2.5-10 nm in the *c*-direction.

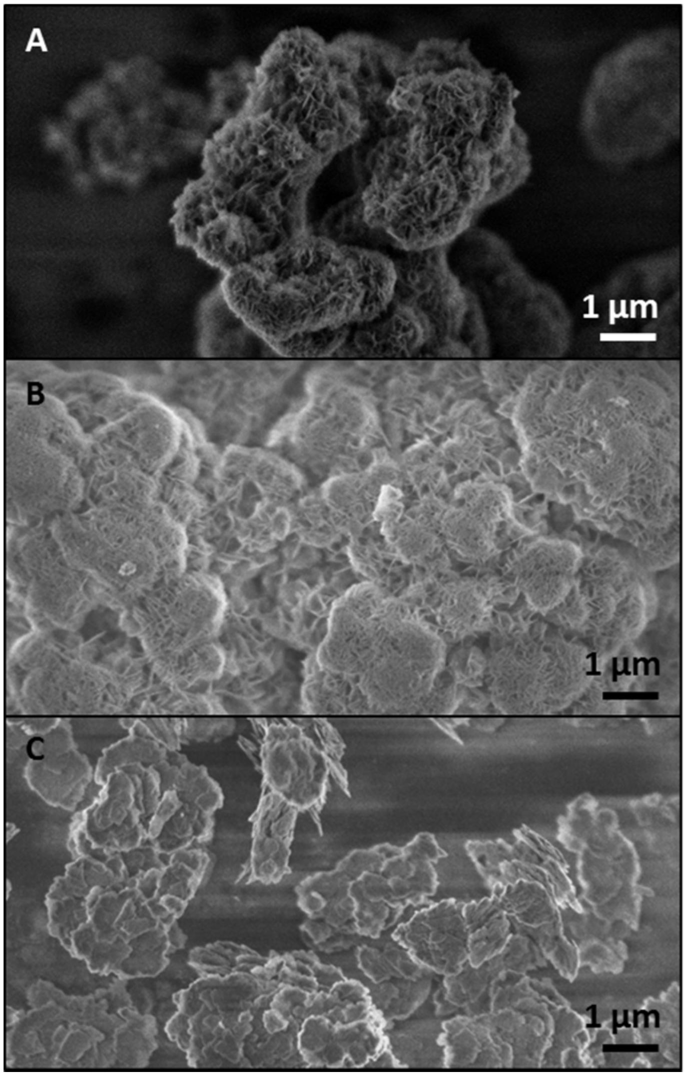

Figure S15. SEM images of calcined MIT-1 (A), MCM-56 (B), and MCM-22 (C). Images of MIT-1 and MCM-56 show large aggregates of lamella with rosette features between 1-5 µm in size. MCM-22 shows aggregates of thicker plates that appear to form aggregates between 0.5-5 µm in size.

4. References

(1) Corma, A.; Diaz, U.; Fornés, V.; Guil, J.; Martinez-Triguero, J.; Creyghton, E. *J. Catal.* **2000**, *191*, 218.

(2) Fung, A. S.; Lawton, S. L.; Roth, W. J. Synthetic layered MCM-56, its synthesis and use. U.S. Patent 5362697, Nov 8, 1994.

(3) Boshoff, J. University of Delaware Nanoporous Carbon Research Initiative, Resources, UDSKIP. http://www.che.udel.edu/research_groups/nanomodeling/resources.html (accessed Aug, 9 2012)

(4) Camblor, M. A.; Corma, A.; Díaz-Cabañas, M.-J.; Baerlocher, C. *J. Phys. Chem. B* **1998**, *102*, 44.

(5) Massiot, D.; Bessada, C.; Coutures, J.; Taulelle, F. *Journal of Magnetic Resonance (1969)* **1990**, *90*, 231.

(6) Zhao, Q.; Chen, W.-H.; Huang, S.-J.; Wu, Y.-C.; Lee, H.-K.; Liu, S.-B. *J. Phys. Chem. B* **2002**, *106*, 4462.

(7) Pines, A.; Gibby, M. G.; Waugh, J. S. *The Journal of Chemical Physics* **1972**, *56*, 1776.

(8) Bennett, A. E.; Rienstra, C. M.; Auger, M.; Lakshmi, K.; Griffin, R. G. *The Journal of Chemical Physics* **1995**, *103*, 6951.

(9) Materials Studio 6.1, Accelrys Inc: San Diego, CA, (2012).

(10) Dauger-Osguthorpe, P.; Roberts, V.A.; Osguthorpe, D.J.; Wolff, J.; Genest, M.; Hagler, A.T. *Proteins: Structure, Function and Genetics* **1988**, *4*, 31-47.

(11) Ch. Baerlocher and L.B. McCusker, Database of Zeolite Structures: http://www.iza-structure.org/databases/
